# Prediction intervals reporting in orthodontic meta-analyses

**DOI:** 10.1093/ejo/cjab037

**Published:** 2021-07-31

**Authors:** Jadbinder Seehra, Daniel Stonehouse-Smith, Nikolaos Pandis

**Affiliations:** 1 Department of Orthodontics, Faculty of Dentistry, Oral & Craniofacial Sciences, King’s College London, Guy’s Hospital, Guy’s and St Thomas NHS Foundation Trust, UK; 2 Department of Orthodontics and Dentofacial Orthopedics, Dental School/Medical Faculty, University of Bern, Switzerland

## Abstract

**Background:**

A prediction interval represents a clinical interpretation of heterogeneity. The aim of this study was to determine the prevalence of prediction interval reporting in orthodontic random effect meta-analyses. The corroboration between effect size estimates with 95% confidence intervals (CIs) and prediction intervals were also explored.

**Materials and methods:**

Systematic reviews (SRs) published between 1 January 2010 and 31 January 2021 containing at least one random effects meta-analysis (minimum of three trials) were identified electronically. SR and meta-analyses characteristics were extracted and prediction intervals, where possible, were calculated. Descriptive statistics and the percentage of meta-analyses where the prediction interval changed the interpretation based on the 95% CI were calculated. Fisher’s exact test was used to examine associations between the study variables and reporting of prediction intervals.

**Results:**

One hundred and twenty-one SRs were included. The median number of SR authors was 5 (interquartile range: 4–6). The reporting of prediction intervals was undertaken in only 19.0% (*N* = 23/121) of meta-analyses. Out of 95 meta-analyses, only in 6 (6.3%, *N* = 6/95) were the 95% CI corroborated by the prediction interval. In 60 meta-analyses (63.3%, *N* = 60/95) despite a 95% CI indicating a statistically significant result, this was not corroborated by the corresponding prediction interval.

**Conclusions:**

Within the study timeframe, reporting of prediction intervals is not routinely undertaken in orthodontic meta-analyses possibly due to a lack of awareness. In future orthodontic random effects models containing a minimum of three trials, reporting of prediction intervals is advocated as this gives an indication of the range of the expected effect of treatment interventions.

## Introduction

The conduct and publishing of systematic reviews (SRs) within the literature has increased exponentially (1, 2). When feasible, these reviews aim to combine the results of individual primary trials in a meta-analysis model to report an overall pooled estimate of the treatment intervention. The models commonly undertaken are the fixed and random effects models. In the fixed effect model a common underlying effect is assumed between the included studies. In contrast, the random effects model allows for heterogeneity between the included studies (3). The source of this heterogeneity between studies maybe a result of differences between study participants and interventions or bias (4, 5). As a consequence, in addition to the reporting of the overall pooled estimate, *P* value, the precision of the estimate [95% confidence interval (CI)], and a quantification of the degree of heterogeneity in the random effects model is also stated. These measures of in between trial heterogeneity include *I*^2^ and *τ*^2^ (6). However, it has been argued that the interpretation of these measures in a clinical context are complex. For instance, *I*^2^ is proportional to the sample size which can be misleading especially when only a few studies are included in the random effects model and interpretation is based on statistical significance. Although fixed effect and random effects meta-analyses are distinct entities, the results are often interpreted in the same manner (7). This problem is nicely illustrated by Higgins *et al.* (8) where the interpretation of two meta-analyses that produce the same pooled estimates and interpreted using only the pooled estimate fail to highlight important differences between the two datasets that could radically alter our conclusions. Chiolero *et al.* (9) provide another example on the interpretation using the CI compared with the prediction interval.

To circumvent these issues, the reporting of prediction intervals has been advocated (10), as these can provide a more clinically meaningful assessment of in between trial heterogeneity in random effects meta-analyses (7, 8, 11). The prediction interval is defined as the interval within which the effect size of a new study would fall if this study were selected at random from the same population of the studies already included in the meta-analysis (12). Prediction intervals can be calculated when there are at least three trials in the meta-analysis. Typically, prediction intervals are expected to be wider than 95% CI of the pooled estimate as they combine both the variance of the summary effect and the heterogeneity hence reflecting the added uncertainty of a future trial (13). A simple prediction interval can be calculated as follows (8):


$$\begin{array}{l} M\pm t_{k-2}\sqrt{t^{2\;}+\;SE{\left(M\right)}^{2}} \end{array}$$

where *M* is the summary mean (pooled estimate) from the random effects meta-analysis, $t_{k-2}$ is the 95% percentile of a *t*-distribution with *k* − 2 degrees of freedom, *k* is the number of studies, *t*^2^ is the estimated between study heterogeneity, and SE(*M*) the standard error of the summary mean.

A 95% CI infers that in 95% of the cases, the mean effect size will fall within the parameters of the random effects meta-analysis diamond. In contrast, a 95% prediction interval indicates that in 95% of the cases, the true effect size of a new study will fall within the meta-analysis prediction interval. Therefore, a CI may indicate a statistically significant result, but this does not necessarily mean it will be supported by the corresponding prediction interval. In other words, despite a CI suggesting a significant treatment effect, this is not in agreement with the prediction interval which may suggest that in some clinical setting the treatment or intervention can be ineffective.

To our knowledge, it is unknown if prediction intervals are routinely reported in orthodontic quantitative SRs. Therefore, the aim of this study was to determine the prevalence of prediction interval reporting in orthodontic random effect meta-analyses involving a minimum of three trials. Furthermore, the corroboration between effect size estimates with 95% CIs and prediction intervals were to be explored.

## Materials and methods

### Eligibility criteria

Orthodontic SRs published between 1 January 2010 and 31 January 2021 were searched.

To be included, the SR should include at least one meta-analysis (random effects model) on either binary or continuous outcomes containing a minimum of three trials, be published in English and report interventional procedures involving human participants. In SRs containing multiple meta-analyses (study or subgroup), the single random effects meta-analysis closely matching the SR aim and objective and containing the most primary trials was selected. Where multiple versions of the same SR existed, the latest version was selected. SRs involving animal or *in vitro* studies were excluded.

### Search and selection of SRs

An electronic database search was undertaken using Medline via PubMed (www.pubmed.ncbi.nlm.nih.gov). One author (JS) performed a literature search of databases using medical subject headings ‘orthodontic’ AND ‘systematic review’ OR ‘meta-analysis’.

All relevant orthodontic SRs published in the Cochrane Library of Systematic Reviews (www.cochranelibrary.com) were also screened. All titles and abstracts were screened by one author (JS). Full-text articles of abstracts fulfilling the inclusion criteria were retrieved and further analysed for eligibility independently by two authors (JS and DSS). Any disagreements in the final SRs were resolved by discussion among the authors (JS and DSS) with the involvement of a third author (NP) if required. However, no disagreements were identified.

### Data extraction

All study characteristics were initially extracted by a single author (JS) and entered into a pre-piloted Microsoft Excel® (Microsoft, Redmond, Washington, USA) data collection sheet (Supplementary Table 1). A second author (DSS) cross-checked the collected data. Any disagreements were resolved by discussion. At the level of the SR the following information was extracted: year of publication; number of authors; continent of corresponding author (Europe, Americas, and Asia or other); PROSPERO registration (no or yes); type of review (Cochrane and non-Cochrane) (no or yes) and whether the authors discussed the relevance of the reported prediction interval estimates within the article (no, yes, and non-applicable). At the level of the selected meta-analysis the following information was extracted: number of primary trials included in the meta-analysis; prediction intervals reported (no or yes); significance of the result (no or yes), reported 95% CIs, the pooled estimate, effect measure, and the *τ*^2^ value. Wherever the *τ*^2^ was provided, the SEs for the pooled estimates were back calculated using the reported CIs (14). Consequently, the prediction interval was calculated for the meta-analyses when it was not reported using the formula shown earlier. For binary outcomes before the calculation of the prediction intervals a logarithmic transformation was applied.

### Statistical analysis

Descriptive statistics and the percentage of meta-analyses where the prediction interval changed the interpretation based on the 95% CI were calculated. Fisher’s exact test and exact logistic regression were used to examine associations between the study variables year of publication, number of authors, continent of corresponding author, PROSPERO registration, type of review, significance of the result and reporting of prediction intervals. A two-tailed *P* value of 0.05 was considered statistically significant. All analyses were performed using Stata statistical software version 16.1 (StataCorp, College Station, Texas, USA) and the R statistical package (Vienna, Austria).

## Results

**Figure 1.**
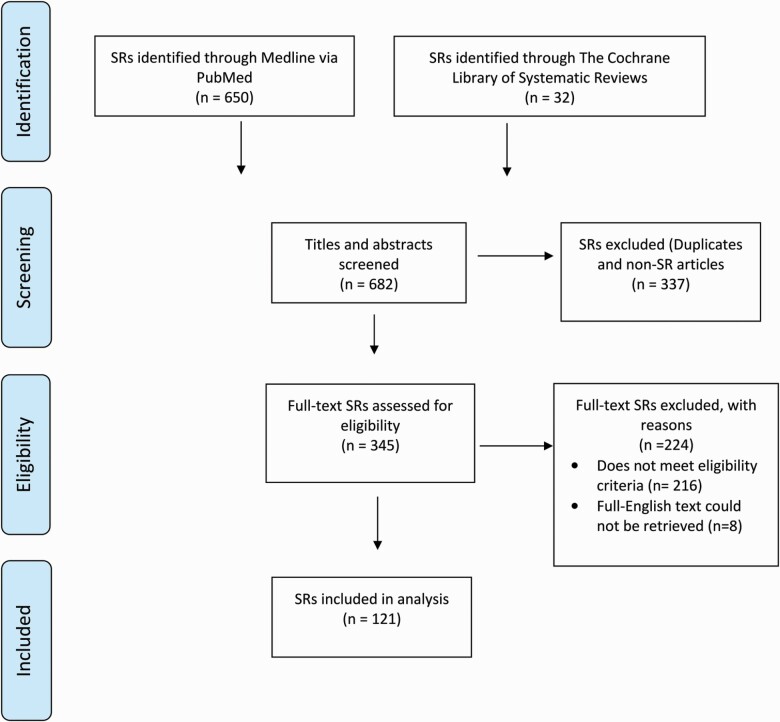
Flow diagram for the identification and selection of systematic reviews.

A total of 121 SRs met the eligibility criteria and were analysed (Figure 1). The median number of authors for the sample was 5 (interquartile range 4–6). Between 2012 and 2021, prediction intervals were infrequently reported and ranged between 0 and 5 per year. Reporting of prediction intervals was evident with corresponding authors based in Europe (41.2%), SR registered with PROSPERO (27.5%), and non-Cochrane reviews (23.0%) (Table 1).

**Table 1.** Study characteristics SRs by reporting of prediction intervals (*N* = 121).

Variable	Not reported *N* (%)	Reported *N* (%)	Odds ratio	95% confidence interval	Fishers exact test *P* < 0.05
Year of publication			0.85*	0.67, 1.08*	0.18*
2012	0 (0.0)	1 (100.0)			
2013	2 (66.7)	1 (33.3)			
2014	5 (83.3)	1 (16.7)			
2015	6 (75.0)	2 (25.0)			
2016	11 (78.6)	3 (21.4)			
2017	18 (81.8)	4 (18.2)			
2018	21 (84.0)	4 (16.0)			
2019	16 (76.2)	5 (23.8)			
2020	17 (89.5)	2 (10.5)			
2021	2 (100.0)	0 (0.0)			
Number of authors			0.55	0.37, 0.82	0.001
Continent of corresponding author					
Europe	30 (58.8)	21 (41.2)	Reference	Reference	
Americas	17 (89.5)	2 (10.5)	0.17	0.04, 0.81	<0.001
Asia or other	52 (100.0)	0 (0.0)	Not estimable	Not estimable	
PROSPERO registration					
No	53 (89.8)	6 10.2)	Reference		
Yes	45 (72.5)	17 (27.5)	3.30	1.12, 11.14	0.03
Type					
Non-Cochrane	92 (80.0)	23 (20.0)	Reference		
Cochrane	6 (100.0)	0 (0.0)	0.50	0.0, 3.66	0.55
Significance of results					
Non-significant	39 (76.5)	12 (23.5)	Reference		
Significant	59 (85.5)	10 (14.5)	0.55	0.19, 1.55	0.31
Not reported	0 (0.0)	1 (100.0)	Not estimable	Not estimable	
Total	98	23			

SRs, systematic reviews.

*Estimation using year as a continuous variable.

Within this sample, the reporting of prediction intervals was undertaken in only 19.0% (*N* = 23/121) of meta-analyses. Despite prediction intervals being calculated, in the majority of these SRs the relevance of these estimates was not discussed in the article (73.9%, *n* = 17/23). Fisher’s exact test showed an association between the reporting of prediction intervals and the following study variables: continent of corresponding author (*P* < 0.001) and PROSPERO registration (*P* = 0.02) (Table 1). The most commonly reported effect measure was the mean difference (64.5%) and the *τ*^2^ was not reported in 26 meta-analyses (21.5%, *N* = 26/121) (Table 2).

**Table 2.** Effect measure and reporting of *τ*^2^ (*N* = 121).

Variable	*N* (%)
Effect measure (*N* = 121)	
Mean difference	78 (64.5)
Standardized mean difference	23 (19.0)
Risk ratio	11 (9.1)
Odds ratio	9 (7.4)
*τ* ^2^ (*N* = 121)	
Reported	95 (78.5)
Not reported	26 (21.5)

**Figure 2.**
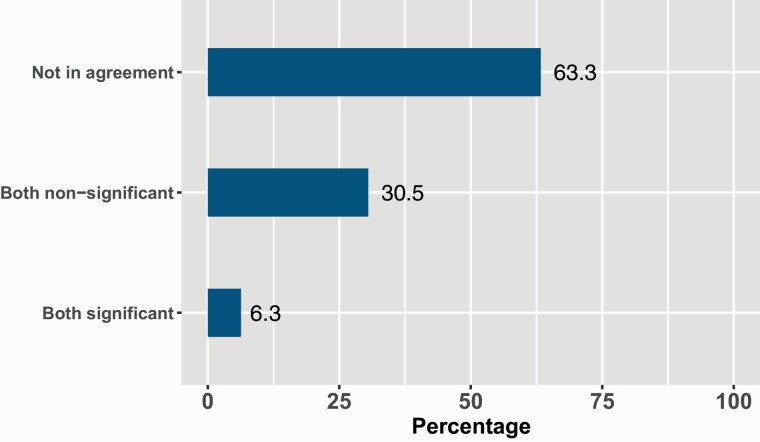
Agreement between confidence intervals and prediction intervals (*N* = 95).

In addition, to the 23 meta-analyses reporting prediction intervals, this estimate was possible to be recalculated for a further 72 meta-analyses based on the reporting of the pooled estimate, 95% CIs, and *τ*^2^ values. In this total cohort of 95 meta-analyses, only in 6 (6.3%, *N* = 6/95) was a significant 95% CI corroborated by the prediction interval. In 29 meta-analyses (30.5%, *N* = 29/95) both the 95% CI and prediction interval were non-significant. However, in 60 meta-analyses (63.3%, *N* = 60/95) despite a 95% CI indicating a statistically significant result, this was not corroborated by the corresponding prediction interval (Figure 2).

## Discussion

Within this sample, prediction intervals were only reported in 19.0% of SRs. The lack of reporting of prediction intervals in orthodontic SRs could be a result of a lack of awareness of this measure of heterogeneity of treatment estimates or attributed to factors at the level of the SR. Prediction intervals are only undertaken in random effects meta-analysis models with a minimum of three trials (13). In the current study, a vast majority of SRs were excluded as they do not meet these requirements which is supported by the fact that the conduct of meta-analyses involving a small number of trials is common in oral health (15, 16). Prior to 2021, Cochrane reviews tended not to report this estimate which supports the observation that prediction intervals were not reported in these reviews (Table 1). However, in the recent update of the Cochrane handbook, the reporting of prediction intervals is now advocated (17). It is well established that SR registration improves the quality of the review (18) and hence it is not a surprise that the reporting of prediction intervals is associated with PROSPERO registration.

A prediction interval provides a predicted range for the true treatment effect in a future study conducted under similar settings. The value of this estimate of heterogeneity in a clinical context is highlighted by the following extract: ‘Meta-analysis of these studies suggested higher odds of bond failures with the Self Etch Primer technique, although the difference failed to reach statistical significance (odds ratio, 1.35; 95% CI, 0.99–1.83). The pooled odds ratio from the random-effects model indicated that the failure risk was 35% higher in the Self Etch Primer group than in the Acid Etch group. The 95% CI indicates that the mean effect size can range from 1% less to 83% greater in the Self Etch Primer group compared with the Acid Etch group, verging on statistical significance (*P* = 0.06). Based on the heterogeneity of the included studies, the prediction intervals indicate that the true effect size of a future trial is likely to range from 0.82 to 2.22’ (19). Importantly, in this example the value of no difference (1 relative difference) is included in the prediction intervals. This trend also appears to be replicated in larger samples of SRs. In the current study, in only six meta-analyses (6.3%, *N* = 6/95) with significant 95% CI there was agreement with the prediction interval. This means the interpretation of future studies conducted in similar settings is not likely to change. In contrast, in 60 meta-analyses (63.3%, *N* = 60/95) despite a 95% CI indicating a statistically significant result, this was not corroborated by the corresponding prediction interval. This means in similar settings no difference is expected between the effects/interventions and there is weak evidence to support the effectiveness of the compared interventions regarding the studied outcomes in a future study. This disparity between estimates of heterogeneity is not unusual. In the assessment of Cochrane SRs, out of 479 statistically significant random effects meta-analyses the prediction interval indicated that in 72.4% SRs the intervention effect could be null or in the opposite direction. Of more concern is that in 20.3% of the 479 meta-analyses the effect could have been completely opposite to that of the meta-analysis (9).

The routine reporting of prediction intervals in addition to the summary effect and its CI has been advocated (10). The rationale for this is that prediction intervals give both an indication of the range of true effects that could be expected in future settings but also the expected effect in the treatment of patients (10). Importantly, the latter could have more impact on health care decisions. However, the interpretation of prediction intervals is associated with certain caveats. Firstly, any conclusions made from the prediction intervals is based on the assumption that *τ*^2^ and estimate of the study effect are both normally distributed. Secondly, imprecision of both *τ*^2^ and the estimated treatment effect as result of the inclusion of few studies which are also small, will lead to imprecise prediction intervals estimates. Lastly, the uncertainty demonstrated by the prediction interval only applies to the uncertainty about the extent to which participants in future studies are similar to those that have been included in the meta-analysis. Clinically, this means that if the patients treated are different to those included in the meta-analysis, then prediction intervals cannot inform clinicians the likely intervention effect in these patients (10).

The representation of prediction intervals within random effect forest plots has been subject to debate (5). A hollow diamond similar to the one used to display the 95% CIs for the average effect has been suggested (8). Extra lines to the left and right end of the effect size diamond have been also postulated (7). However, these graphical proposals of the prediction intervals may lead to confusion resulting in clinicians unable to distinguish between prediction intervals and CIs which are distinct entities (5). The CIs describe the precision of the mean effect size, and the prediction interval reflects the dispersion of the true effect sizes of the new studies (13). An alternative suggestion is the use of a rectangle which is included in a separate row within the forest plot (5, 11).

All titles and abstracts were screened by only one author which may have introduced selection bias. However, the aim to bring awareness to the problem and not to provide exact estimates of prediction interval reporting. Furthermore, as only two databases were searched and SRs limited to those published in English, again SRs that met the eligibility criteria may have not been identified and included hence the reported results could be under-estimating the issue of reporting prediction intervals. Despite this 121 SRs met the eligibility criteria which represents a significant sample size highlight the issue of prediction interval reporting in orthodontic SRs.

## Conclusions

Within the study timeframe, reporting of prediction intervals is not routinely undertaken in orthodontic meta-analyses possibly due to a lack of awareness. In future orthodontic random effects models containing a minimum of three trials, reporting of prediction intervals is advocated as this gives an indication of the range of the expected effect of treatment interventions.

## Supplementary material

Supplementary material is available at *European Journal of Orthodontics* online.

Supplementary Table 1. Data extraction form.

cjab037_suppl_Supplemental_Table_1

## Data Availability

The data underlying this article are available in the article and in its online supplementary material.
